# Genome-Scale Transcriptome Analysis in Response to Nitric Oxide in Birch Cells: Implications of the Triterpene Biosynthetic Pathway

**DOI:** 10.1371/journal.pone.0116157

**Published:** 2014-12-31

**Authors:** Fansuo Zeng, Fengkun Sun, Leilei Li, Kun Liu, Yaguang Zhan

**Affiliations:** 1 State Key Laboratory of Tree Genetics and Breeding (Northeast Forestry University), Harbin 150040, China; 2 College of Life Science, Northeast Forestry University, Harbin 150040, China; Center for Cancer Research, National Cancer Institute, United States of America

## Abstract

Evidence supporting nitric oxide (NO) as a mediator of plant biochemistry continues to grow, but its functions at the molecular level remains poorly understood and, in some cases, controversial. To study the role of NO at the transcriptional level in *Betula platyphylla* cells, we conducted a genome-scale transcriptome analysis of these cells. The transcriptome of untreated birch cells and those treated by sodium nitroprusside (SNP) were analyzed using the Solexa sequencing. Data were collected by sequencing cDNA libraries of birch cells, which had a long period to adapt to the suspension culture conditions before SNP-treated cells and untreated cells were sampled. Among the 34,100 UniGenes detected, BLASTX search revealed that 20,631 genes showed significant (E-values≤10^−5^) sequence similarity with proteins from the NR-database. Numerous expressed sequence tags (i.e., 1374) were identified as differentially expressed between the 12 h SNP-treated cells and control cells samples: 403 up-regulated and 971 down-regulated. From this, we specifically examined a core set of NO-related transcripts. The altered expression levels of several transcripts, as determined by transcriptome analysis, was confirmed by qRT-PCR. The results of transcriptome analysis, gene expression quantification, the content of triterpenoid and activities of defensive enzymes elucidated NO has a significant effect on many processes including triterpenoid production, carbohydrate metabolism and cell wall biosynthesis.

## Introduction

Nitric oxide (NO) is a noxious free radical gas, which in the late 1980s was discovered to exist physiologically in mammalian systems. Notably, the idea that a simple gas could act as a messenger revolutionized the understanding of signal transduction [1], [2]. NO could be produced by nitric oxide synthase (NOS) and nitrate reductase (NR) pathways within plants [3], [4]. Recently, NO was shown to mediate diverse plant physiological processes such as germination, root growth, flowering, stomatal closure, and resistance to biotic as well as abiotic stresses [5]–[8]. NO is also a key molecule in signal transduction pathways that initiates secondary metabolite biosynthesis in plants [9], [10]. Parani et al (2004) showed that NO modulated the expression of a substantial number of genes at the transcriptional level in *Arabidopsis thaliana*. Hierarchical clustering revealed 162 genes showing a dose-dependent increase in signal from 0.1 mM SNP to 1.0 mM SNP treatment [11]. Although the evidence supporting NO as a mediator of plant physiological processes continues to grow, its functions at the molecular level remain poorly understood.

Genome-scale transcript analysis aims to capture an unbiased view of the complete RNA transcript profile of a species, allowing the transcriptional level of each gene in a given tissue at a given point in its life cycle to be monitored [12]. Transcriptome analysis is also an efficient means of analyzing the overall transcript levels within specific tissues and organs, which not only generates large numbers of expressed sequence tags (ESTs) but also builds gene expression profiles [13]. The gene expression level can be determined by its relative reads per kilobases per million reads (RPKM) values, which is a measurement of the number of RNA-sequencing reads mapped to the constitutive exons of a certain gene and reflects whether a gene is transcribed and its relative abundance. Therefore, the construction and analysis of a transcriptome can provide rapid and improved understanding of gene expression and aid in the discovery of novel genes involved in various cellular processes [14].

Birch (*Betula platyphylla* Suk.) is a broad-leaved pioneer tree species native to eastern Asia, and it grows and regenerates quickly in disturbed habits. Plant cell cultures are useful systems for the production of specific and valuable secondary metabolites. Triterpenoids such as oleanolic acid, betulin, and betulinic acid are pharmaceutical secondary metabolites with antibacterial, antiviral, and antitumor properties, which can be extracted from *B. platyphylla* Suk [15]–[17].

The objective of this study was to investigate the role of NO at the transcriptional level in *B. platyphylla* cells by genome-scale transcriptome analysis. Here, we present a *de* *novo* transcriptome assembly of birch cells treated with sodium nitroprusside (SNP) using Solexa data. Data were collected by sequencing cDNA libraries of birch cells, which had a long period to adapt to the suspension culture conditions before SNP-treated cells and untreated cells were sampled. We specifically examined gene expression dynamics in this species in response to SNP treatment and identified a core set of NO-related transcripts. The acquired information should facilitate attempts to elucidate the response of secondary metabolites when NO is present. We believe that this transcriptome analysis, combined with observations of cellular triterpenoid content, and activities of antioxidant defense enzymes, will shed light on the diverse roles of NO that affect plant physiology.

## Materials and Methods

### Birch Cell Culture

*B. platyphylla* cell was cultivated on optimized Nagata–Takebe medium supplemented with 0.1 mgL^−1^ 6-benzyladenine, 0.01 mgL^−1^ thidiazuron, and 20 gL^−1^ sucrose according to our previous research [18]. The pH of the medium was adjusted to 5.5–6.0 and the medium was then sterilized by autoclaving at 121°C for 20 min. The suspension culture (100 mL) was maintained in 250-mL Erlenmeyer flasks incubated on a rotary shaker (110 rpm) at 25°C, and inoculated with 4.0 g fresh weight of 8-day-old cell suspension cultures. Illumination was regulated to provide 14 h of light (photophase 06:00–20:00 h) via fluorescent tubes (a combination of Osram Fluoro and Osram Daylight types) with a photon flux density (400–700 nm) and intensity of 2000 lux.

### RNA Extraction, Quality Determination, and Real-Time qRT-PCR

Cells (0.2 g) were collected by filtration from the culture medium and frozen in liquid nitrogen. Total RNA was isolated using the CTAB-based method and treated with RNase-free DNase I (Takara, Dalian, China) to remove genomic DNA according to the manufacturer’s protocols. After DNase treatment, only the RNA itself was amplified with the same primers to control the absence of DNA in the RNA preparation. The quality of the RNA was verified on an 0.8% agarose gel by using standard procedures. RNA (2.5–5 µg) was loaded onto the gels, and it showed a sharp distinction at the small side of both the 18S and 28S rRNA bands. The absorbance ratios (260 nm/280 nm) of the RNA samples dissolved in 10 mM Tris (pH 7.6) ranged from 1.9 to 2.1. The integrity of RNA samples was examined with an Agilent 2100 Bioanalyzer, and their RIN (RNA integrity number) values ranged from 8.6 to 10.0, with no sign of degradation. One microgram of total RNA was reverse-transcribed into first-strand complementary DNA (cDNA) by using the reverse transcriptase M-MLV (Promega, Medison, USA) with oligo-(dT) primers, according to the manufacturer’s protocols. To verify the transcriptome analysis results, quantitative RT-PCR was performed to quantify the expression levels of the target genes. Quantitative RT-PCR was performed using a Bio-Rad iCycler Real-Time PCR machine. Actin was used as an internal control [19], [20]. The sequences of primers are listed in S1 Table. The PCR reaction was carried out in a final volume of 20 µL, containing 2 L cDNA, 10 µL of SYBR Premix Ex Taq II (Takara), 0.4 µL ROX Reference Dye II (50×), 0.8 µL each of forward and reverse primers. DNA was amplified with an initial denaturation step of 30 s at 94°C, followed by for 40 cycles consisting of 95°C for 30 s, 60°C for 34 s, and 72°C for 1 min. The melting curves were generated (15 s at 95°C, 1 min at 60°C, 15 s at 95°C) after the final PCR cycle. The relative gene expression levels of treatments were compared with the controls by the 2^−ΔΔCt^ method. For the control group, an equal volume of sterile water instead of SNP was added. All measurements were performed in triplicate.

### NO-Related Chemical Reagents, Screening for the Optimal Concentration of SNP, and Treatment Process

After suspended cells were cultured for 8 days, the SNP (Sigma, St. Louis, MO, USA) was added into the culture medium at final concentrations of 0.01, 0.1, 1, and 10 mM respectively. For the controls, the SNP was replaced with an equal volume of sterile water. The cells with 0.01, 0.1, 1, or 10 mM SNP were harvested at 6, 12, 24, 48, 72, 120 and 168 h. The collected cells were then used to measure the total concentration of oleanolic acid. SNP-treated cells (1 mM) and control cells were harvested after incubation for 12 h. Then, the collected cells were used for transcriptome analysis. To verify our results from transcriptome analysis, NO-specific scavenger 2-(4-carboxyphenyl)-4, 4,5,5-tetramethylimidazoline-1-oxyl-3-oxide (cPTIO) (Sigma, St. Louis, MO, USA) was used in the experiments at the final concentration of 150 µM. Eight-day-old cells were pretreated with cPTIO 20 min prior to SNP treatment. The controls received only the vehicle water. The concentrations of NO-related chemical reagents were selected based on previous reports and the results of our preliminary experiments [21], [22]. All determinations were carried out in triplicate.

### Illumina Library Preparation and Solexa Sequencing

For cDNA synthesis and Solexa sequencing, mRNA was isolated from total RNA (20 µg) by using poly-T oligo-attached magnetic beads (Illumina, San Diego, CA, USA) and sheared to small fragments using divalent cations (Illumina) at 94°C for 5 min. cDNA was synthesized using random primers and mRNA fragments as templates. Three paired-end cDNA libraries with 200-bp insert sizes were constructed, and then, the cDNA was sequenced using an Illumina Genome Analyzer (Illumina) according to the manufacturer’s protocols.

### Data Analysis

The reads obtained were randomly clipped into 17-bp K-mers for assembly using de Bruijn graph and SOAPdenovo software (version 1.04) with standard parameters and steps [23]. Briefly, SOAPdenovo first combined the clean reads with 16-mer (K017 bp, do not set the “–R” and “–M” option) length of overlap to form longer fragments without N (i.e., contigs). Subsequently, the reads were mapped back to the contigs. From the paired-end reads, it is possible to detect not only contigs from the same transcript but also the distances between these contigs. Furthermore, SOAPdenovo connects contigs from the same transcript, using N to represent unknown sequences between the two contigs, thus forming scaffolds. Paired-end reads are used again for gap filling within scaffolds to obtain sequences that have the fewest Ns and cannot be extended at either end. To obtain distinct gene sequences, the scaffolds were clustered using TGI Clustering tools; these are defined as UniGenes [24]. To determine the gene abundance, the RPKM value of each gene was calculated as described by Mortazavi and Wang [14], [25].

Individual tentatively unique genes (TUGs) were subjected to BLASTX analysis against the protein databases of NR, Swiss-Prot, KEGG, and COG of NCBI to search for similarity. TUGs with a BLASTX E-value >10^−5^ were discarded for functional annotations. Functional annotations using gene ontology (GO) terms were analyzed by the Blast2GO program [26]. After obtaining GO annotations for all UniGenes, WEGO software was used [27] to perform GO functional classification for all UniGenes according to their molecular functions, involvement in biological processes, and cellular components. Furthermore, the conserved functional motifs of TUGs were also analyzed with InterProScan (http://www.ebi.ac.uk/interpro/scan.html) using default parameters.

### Measurement of Total Triterpenoid Content, Oleanolic Acid Content, Intracellular O_2_^−^, and Antioxidant Enzyme Activities

The total triterpenoid content was determined by spectrophotometry [28] at 551 nm, and the results are expressed as milligrams per gram dry weight (DW). The oleanolic acid content was measured by high-performance liquid chromatography (HPLC) under the following conditions: sample volume of 20 µL, mobile phase was 9 1 v/v acetonitrile-water, flow rate of 1 mL/min, column temperature of 25°C, and detection wavelength of 210 nm. Peroxidase (POD) activity was tested according to the rate of guaiacol oxidation. The POD assay mixture contained 0.1 M phosphate buffer (pH 6.1), 4 mM guaiacol as donor, 3 mM H_2_O_2_ as substrate, and 1.0 mL crude enzyme extract. The total reaction volume was 3.0 mL. The rate of change in absorbance at 420 nm was measured, and the enzyme activity was expressed as the difference in absorbance (OD). Ascorbate peroxidase (APX) activity was measured according to the method of Nakano and Asada [29]. The level of superoxide anion (O_2_^−^) was measured after trichloromethane extraction by detecting the absorbance at 530 nm with the UV spectrophotometer [30].

### Statistical Analysis

All experiments were repeated three times. The data (mean ± standard error) obtained were statistically analyzed using SPSS version 19.0. Data from experiments were analyzed using a Student’s *t*-test for simple comparisons between each treatment and its control, and a two way ANOVA with post-hoc Tukey test for the multiple comparisons between means. Differences at P<0.05 were considered statistically significant.

## Results and Discussion

### Optimization of SNP Concentration

**Figure 1:**
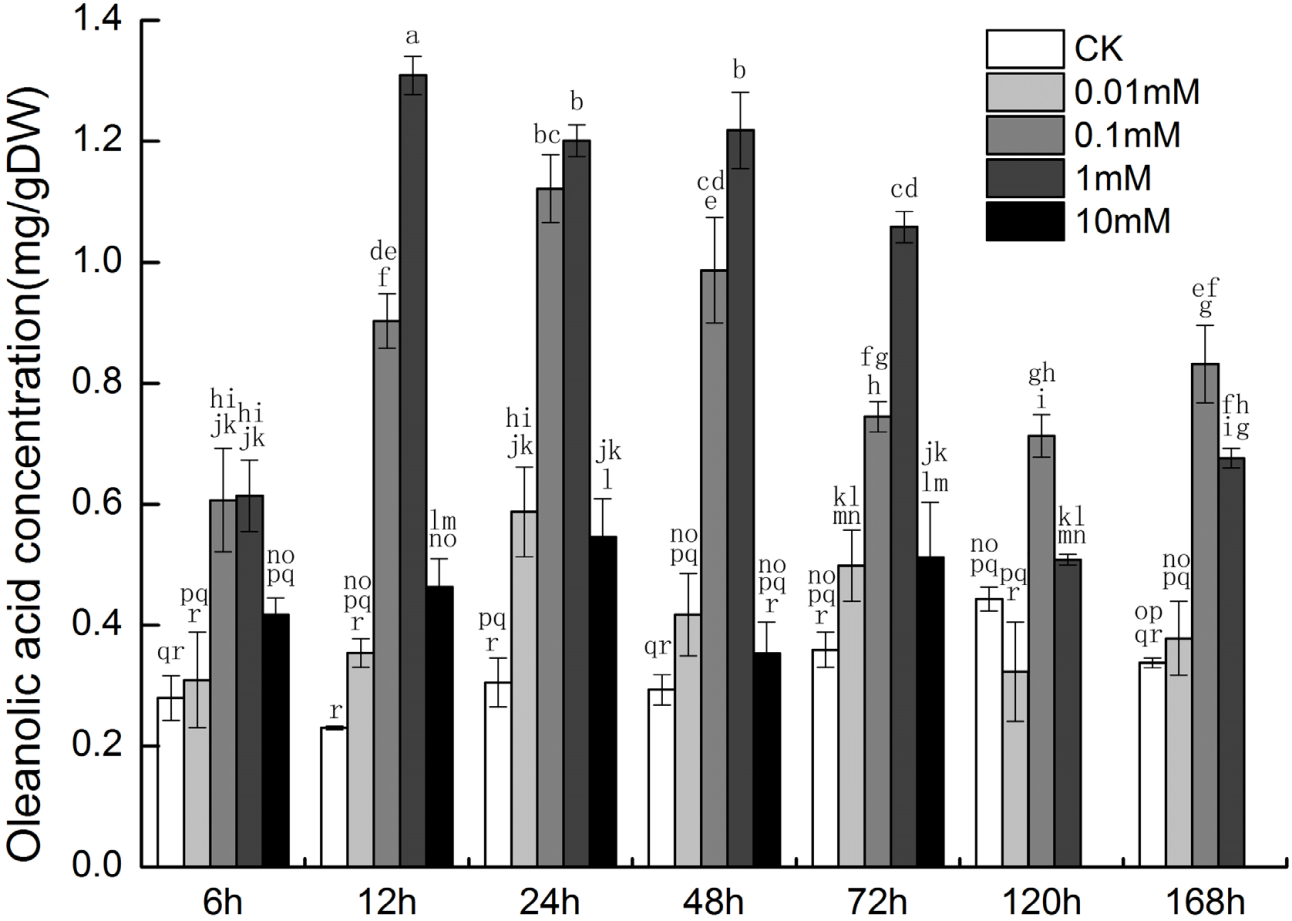
Changes in oleanolic acid content among the control (CK), 0.01 mM, 0.1 mM, 1 mM, and 10 mM SNP groups at different times. Different letters represent significant difference (P<0.05).

The oleanolic acid content increased in a time- and dose-dependent manner when cells were treated with 0.01, 0.1 and 1 mM SNP within 72 h. Oleanolic acid concentrations were observed to be higher in the 1-mM SNP treatment group than that in the other groups, from 12 h to 72 h (Fig. 1). Therefore, 1 mM was used as the optimal concentration of SNP to investigate the effect of exogenous NO in regulating triterpenoid synthesis.

### *De novo* Assembly and Quantitative Assessment of Illumina ESTs

In order to obtain a comprehensive view of the changes in gene expression patterns in response to NO, transcriptome analyses were performed. A total of 12,570,361 reads were obtained by Solexa analysis, containing 6.21 Gb. The percentage of N in the total nucleotide (nt) count was 0.54%, and the percentage of GC in the transcriptome of the birch cell was 39.07%. The high-quality reads were subjected to cluster analysis using the SOAPdenovo program. First, all reads were grouped into contigs. In total, 229,575 contigs were generated, with a total length of 71,768,587 nt and an average length of 312 nt. Nearly 50% of contigs were between 100 and 200 nt. The paired-end contig sequences were grouped into scaffolds, which were grouped into TUGs, and reads were used for gap filling. The longest sequence was 39,247 nt. In total, 104,449 TUGs (>150 bp) were generated, with an average length of 537 bp. Finally, we identified a total of 34,100 high-quality UniGene sequences.

### Functional Annotation of Unique Genes

**Figure 2:**
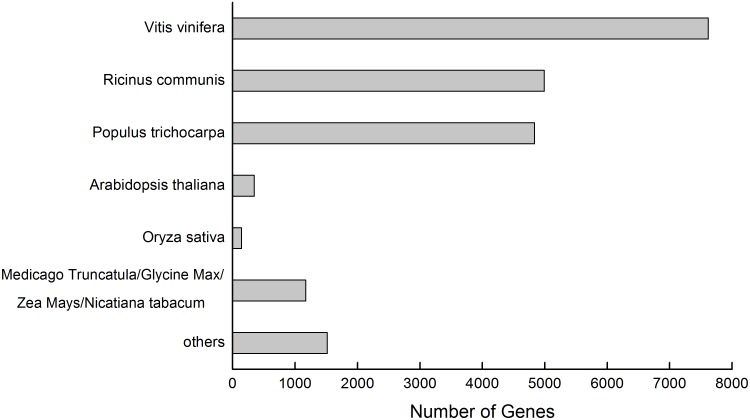
Distribution of UniGenes among different plant species.

**Figure 3:**
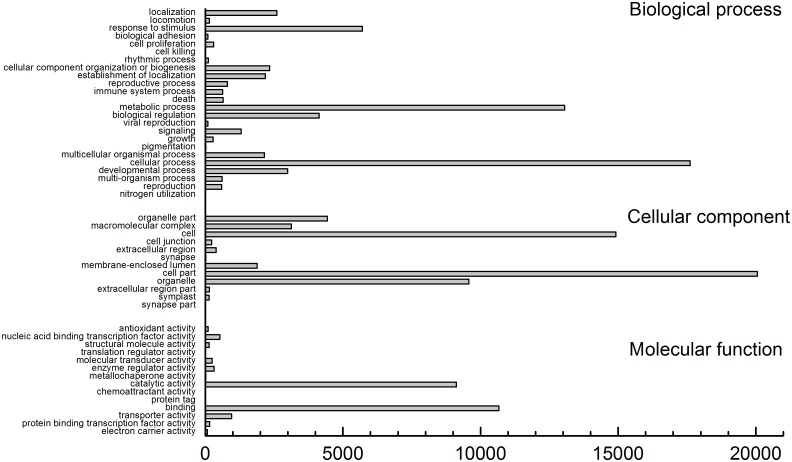
GO categories for the birch cell UniGenes. The percentage and total number of UniGenes in each category is shown.

A BLASTX search was used for the functional annotation of the genes. Among the 34,100 UniGenes, 20,631 genes showed significant (E-values≤10^−5^) sequence similarity with proteins from the NR-database. The proteins that most highly matched the putative birch proteins were derived from various plants, particularly *Vitis vinifera* L. (7621), *Ricinus communis* L. (4994), and *Populus* sp. (4836) (Fig. 2). However, there were relatively few genes that matched proteins from model plants such as *Arabidopsis thaliana* L. (346) and *Oryza sativa* L. (144) (Fig. 2). These results suggest that birch shares a high degree of protein sequence similarity with *V. vinifera*. Using the best hits found by BLASTX, an inferred putative function was assigned to the sequences and they were sorted into major functional categories (Fig. 3). These genes were classified into three main functional groups: genes involved in biological processes, genes encoding cellular components, and genes involved in molecular functions.

### Transcript Differences between Control and SNP-Treated Cells

**Figure 4:**
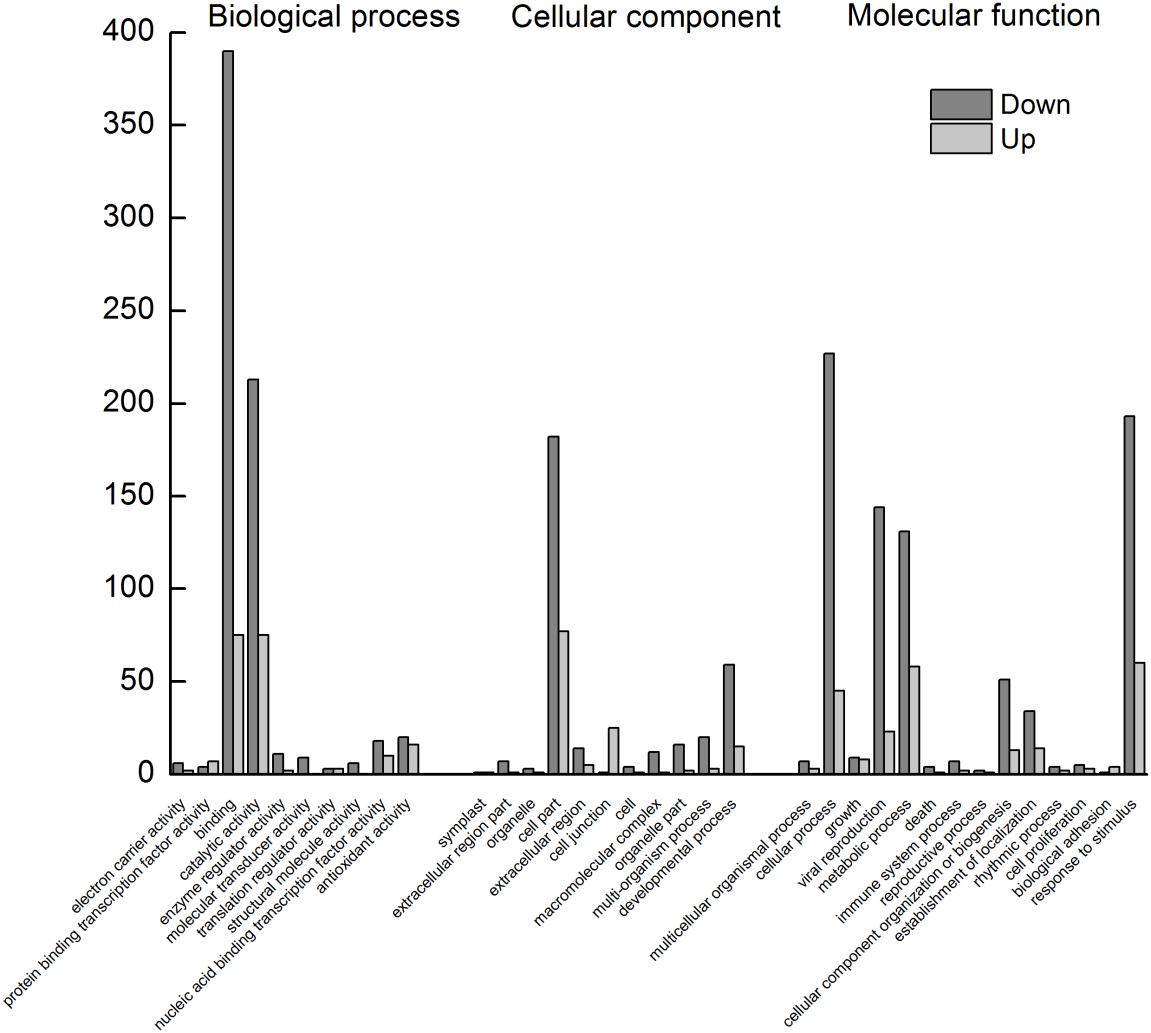
GO categories of differentially expressed ESTs.

According to the applied criteria (two-fold or greater change and *P*<0.001), 1,374 ESTs were identified as differentially expressed between the 12-h SNP-treated cells and control cells samples: 403 up-regulated and 971 down-regulated. This 12-h SNP treatment had apparently modified the expression of almost 4.03% of the total UniGenes. Therefore, it is obvious that birch cells respond to SNP treatment by moderate reprogramming of its transcriptome. We have clustered the differentially expressed genes according to known functions. Genes can have more than one particular function assigned, so some genes can appear in more than one group. Additionally, a GO analysis was performed using Blast2GO to determine gene enrichment and overrepresentation in the three categories: molecular function, biological processes, and cellular components (Fig. 4). Taken together, these experiments were used to analyze the specific differences in transcript expression induced by NO.

### Protection against Reactive Oxygen Species (ROS)

ROS are chemically reactive molecules that contain oxygen, such as superoxide anion radical (O_2_^−^) and hydrogen peroxide (H_2_O_2_). These highly reactive chemicals are natural by-products of aerobic metabolism, and ROS overproduction is toxic as it causes damage to carbohydrates, lipids, proteins, and DNA [31]. The data from our transcriptome analysis revealed that transcript abundance of genes coding for different components of the ROS scavenging machinery does differ dramatically between control cells and SNP-treated cells.

A total of 30 genes encoding proteins with antioxidant properties were up-regulated during NO treatment. These predicted proteins were glutathione *S*-transferases (GST) 1, 2, and 3, thioredoxin peroxidase (TPx), superoxide dismutase (SOD), ascorbate peroxidase (APX), and peroxidase (POD). These up-regulated genes also include four NADP-dependent oxidoreductases, a transcription factor LONG HYPOCOTYL 5 (HY5) and thioredoxin (Trx), all of which play a role in the active synthesis of ROS or in antioxidant defense [32], [33].

**Figure 5:**
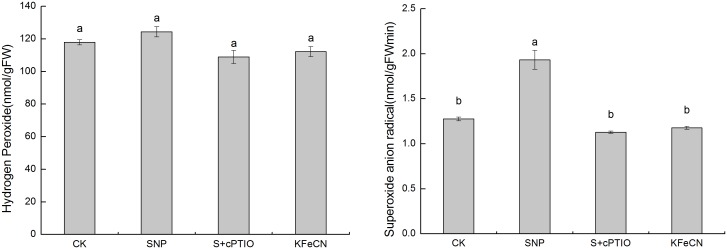
Changes in H_2_O_2_ and O_2_^−^ contents among the control (CK), SNP, KFeCN, and SNP + cPTIO groups (S + cPTIO). Different letters represent significant difference (P<0.05).

**Figure 6:**
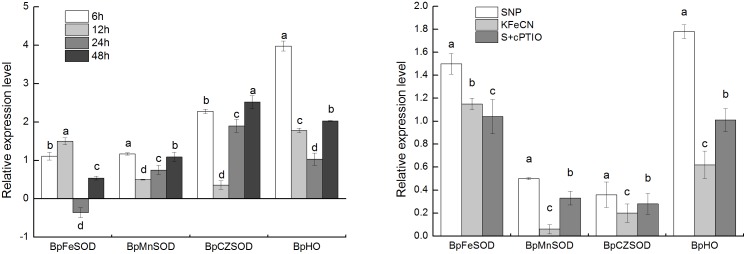
Relative expression levels of antioxidant genes in the groups treated with 1 mM SNP for 6, 12, 24, and 48 h (left); relative expression levels of antioxidant genes in the SNP, KFeCN, and SNP + cPTIO groups (S + cPTIO) (right). Different letters represent significant difference (P<0.05).

**Figure 7:**
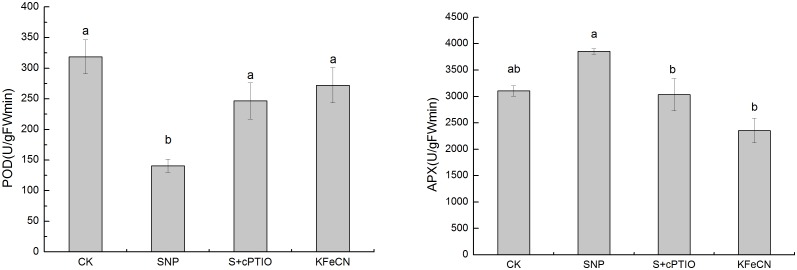
Changes in antioxidant enzyme activities, including POD and APX among the control (CK), SNP, KFeCN, and SNP + cPTIO (S + cPTIO) groups. Different letters represent significant difference (P<0.05).

The levels of intracellular H_2_O_2_ and O_2_^–^ were analyzed among the control, SNP + cPTIO and KFeCN groups at 12 h (Fig. 5). The content of O_2_^–^ in cells treated with 1 mM SNP were indeed higher than the control group. There was no significant difference in the contents of intracellular H_2_O_2_ among the control, SNP + cPTIO and KFeCN groups. To verify our results from transcriptome analysis, qRT-PCR was also performed first to quantify the expression levels of *BpFeSOD* (Fe superoxide dismutase), *BpMnSOD* (Mn superoxide dismutase), *BpCZSOD* (copper/zinc superoxide dismutase), and *BpHO* (heme oxygenase) (Fig. 6). The NO scavenger (SNP + cPTIO group) and SNP structural analogue (KFeCN group) treatments were used as controls to reveal the response of these genes to NO signal. In addition, the gene expression dynamics of birch cells in response to SNP treated after 6, 12, 24, and 48 h were analyzed to define the time-dependence for selected genes. The results showed that four genes were significantly up-regulated after the 6-h SNP treatment. Except for *BpFeSOD* after 24 h SNP treatment that was expressed at a lower level than that of the control, expression of other genes was higher than that of the control at different time points in response to SNP. Our qRT-PCR analysis indeed indicated that several SOD genes were up-regulated in cells by NO treatment. Enzyme activity analysis was performed to examine the increased levels of POD and APX in cells treated with NO (Fig. 7). Consistent with mRNA levels, enzyme activity analyses indicated that APX was expressed predominantly in cells treated by NO. Enzyme activity of POD was lower upon SNP treatment at 12 h, but it was higher than control from 24 h to 168 h (data not shown). Taken together, the results from qRT-PCR and enzyme activity analyses were consistent with our initial transcriptome analysis. Therefore, it is concluded that NO could cause oxidative stress in these cells, and regulate transcription of genes encoding antioxidant enzymes.

In addition, the genes related to plant–fungus interactions are overrepresented in the genes preferentially expressed in cells treated by NO. These include two genes encoding Pathogen-related protein (PR1C, PRPX), a gene encoding Allene oxide synthase, an enzyme responsible for salicylic acid (SA) biosynthesis, and a gene coding for indole-3-acetic acid amido synthetases (GH3.6). Members of the GH3 family encode enzymes that catalyze adenylation of both Indole-3-acetic acid (IAA) and SA [34]. Overexpression of GH3 induces disease resistance [35], [36]. Overall, we conclude that the transcriptome in cells treated by NO resembles that of biotic stress responses. It is possible that biotic defense responses are activated developmentally independent of exposure to NO.

The enzymatic ROS scavenging system includes superoxide dismutase (SOD), catalase (CAT), ascorbate peroxidase (APX) and peroxidase (POD). FeSOD, MnSOD, and CZSOD are the three isozymes of SODs in plants [37]. Plant heme oxygenases (HOs) regulate the biosynthesis of phytochrome, which accounts for photo-acceptance and -morphogenesis. Recent studies have demonstrated that plant HOs also regulate many other physiological processes including response to environmental stimuli and ROS [38].

### Carbohydrate Metabolism and Cell Wall Biosynthesis

One characteristic GO category found in cells treated with NO, is carbohydrate metabolism and cell wall biosynthesis. The expression of beta-d-xylosidase (*BXL1, 2*), Aldose 1-epimerase (*GALM*), UDP-glucose 4-epimerase (*GALE2*), Sucrose synthase 2 (*SUS2*), which are involved in carbohydrate metabolism were enhanced by NO treatment. In the study, genes for cellulose synthase (*CESA8*), cellulose synthase-like proteins (*CSLE6*), xyloglucan endotransglycosylases (*XETs*), caffeic acid 3-O-methyltransferase (*COMT1*), and 4-coumarate–CoA ligase-like 9 (*4CLL9*), and inositol oxygenase 4 (*MIOX4*) are more abundantly expressed in cells treated with NO as compared to control cells. MIOX, which is a key enzyme in UDP-glucuronic acid biosynthesis, is involved in cell wall synthesis.

**Figure 8:**
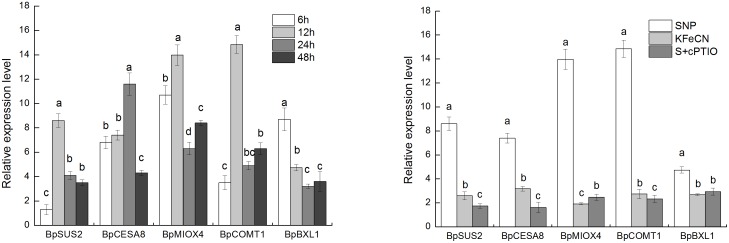
Relative expression levels of genes related to cell wall synthesis in the groups treated with 1 mM SNP for 6, 12, 24, and 48 h (left). Relative expression levels of genes related to cell wall synthesis in SNP, KFeCN, and SNP + cPTIO (S + cPTIO) groups (right). Different letters represent significant difference (P<0.05).

To verify our results from transcriptome analysis, qRT-PCR was performed to quantify the expression levels of *BpSUS2*, *BpCESA8*, *BpMIOX4*, *BpCOMT1,* and *BpBXL1* (Fig. 8). The NO scavenger (SNP + cPTIO group) and SNP structural analogue (KFeCN group) treatments were used as controls to reveal the response of these genes to NO exposure. The results showed the expression levels of *BpSUS2, BpCESA8*, *BpMIOX4, BpCOMT1,* and *BpBXL1* in the SNP group was higher than those in the KFeCN and SNP + cPTIO groups. The gene expression dynamics of birch cells in response to SNP treatment for 6, 12, 24, and 48 h were also analyzed to define the time-dependence for selected genes. The results showed that five genes were significantly up-regulated after 6-h SNP treatment. The expression levels of *BpSUS2, BpMIOX4, and BpCOMT1* were highest at 12 h SNP treatment. The expression of other genes was also higher in SNP-treated cells as compared to control cells. Our qRT-PCR did indeed indicate that these genes were up-regulated in cells as a result of NO treatment.

### Terpenoid Biosynthesis

Another enriched GO category in the genes preferentially expressed in cells treated by NO, are those of secondary metabolism, especially for the terpenoid biosynthesis pathway. Genes for chalcone synthase (CHS), isoflavone reductase, isoflavone 2′-hydroxylase are expressed most abundantly in the cells treated by NO. The expression of 1-deoxy-d-xylulose-5-phosphate synthase (DXS), 3-hydroxy-3-methylglutaryl-coenzyme A reductase (HMGR), cytochrome P450; secologanin synthase (SLS), cycloartenol synthase (CAS), squalene synthetase (SQS), β-amyrin synthase (AMS), allene oxide synthase (AOS), which are involved in triterpenoid synthesis, are abundantly expressed in cells exposed to NO as compared to control cells.

**Figure 9:**
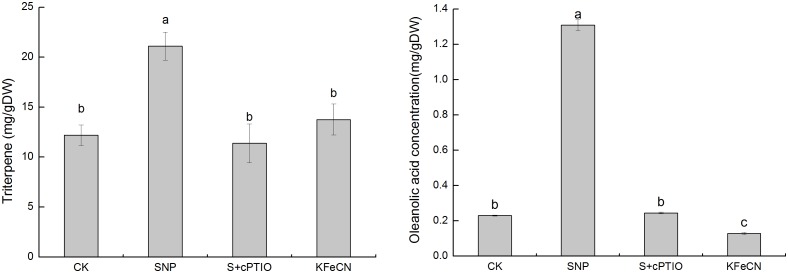
Changes in total triterpene and oleanolic acid content in the control (CK), SNP, KFeCN, and SNP + cPTIO (S + cPTIO) groups. Different letters represent significant difference (P<0.05).

**Figure 10:**
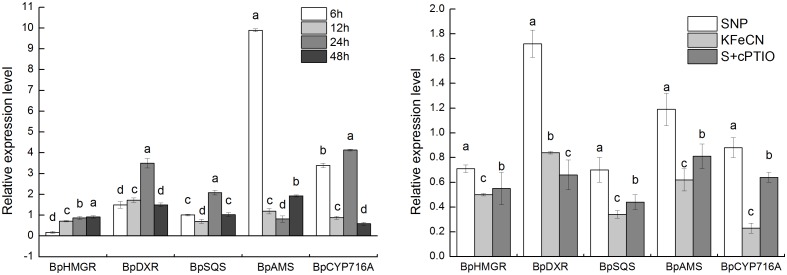
Relative expression levels of genes related to triterpene synthesis in the groups treated with 1 mM SNP for 6, 12, 24, and 48 h (left). Relative expression levels of genes related to triterpene synthesis in the SNP, KFeCN, and SNP + cPTIO (S + cPTIO) groups (right). Different letters represent significant difference (P<0.05).

The oleanolic acid and triterpene contents in KFeCN, and SNP + cPTIO groups were significantly reduced when compared to the SNP group, which suggested that NO could promote triterpenoid synthesis (Fig. 9). The expression of some key genes in the triterpenoid synthesis were analyzed by qRT-PCR to verify our results from transcriptome analysis. The expression levels of *BpHMGR*, *BpDXR*, *BpSQS*, *BpAMS,* and *BpCYP716A* in the SNP group were 2.73, 4.99, 3.92, 3.41 and 9.00 times higher, respectively, than the corresponding levels in the KFeCN group (Fig. 10). The expression levels of these genes were significantly reduced in the SNP + cPTIO group, which suggested that NO could mediate triterpenoid synthesis by regulating the expression of the key enzymes. The time-dependence for the key genes in the triterpenoid synthesis was also revealed by the gene expression dynamics of birch cells in response to SNP treatment. The results showed that five genes were significantly up-regulated after the 6-h SNP treatment. The expression levels of *BpAMS* and *BpCYP716A* were highest at 6 h of SNP treatment. The expression of *BpDXR* and *BpSQS* were highest after 24 h of SNP treatment.

The isopentyl diphosphate and dimethylallyl diphosphate are the common precursors for triterpenoid biosynthesis, which come from mevalonic pathway and methylerythritol 4-phosphate pathway [39]–[41]. The acetyl coenzyme A could form mevalonate under the catalysis of 3-hydroxy-3-methylglutaryl coenzyme A reductase (HMGR) and the formation of methylerythritol 4-phosphate is catalyzed by deoxy-xylulose phosphate isomerase (DXR) [42], [43]. 2,3-Oxidosqualene can be synthesized in the presence of squalene epoxidase (SQS) and then formation of oleanolic acid is catalyzed by β-amyrenol and cytochrome P450 enzyme (CYP716A) [44]. Therefore, HMGR, DXR, SQS, AMS, and CYP716A are key enzymes in the triterpenoid biosynthesis pathway. These results clearly indicated that NO is a key molecule in signal transduction for secondary metabolite biosynthesis in plants, which is similar to results found in other studies [9], [10].

### Growth Regulation

**Figure 11:**
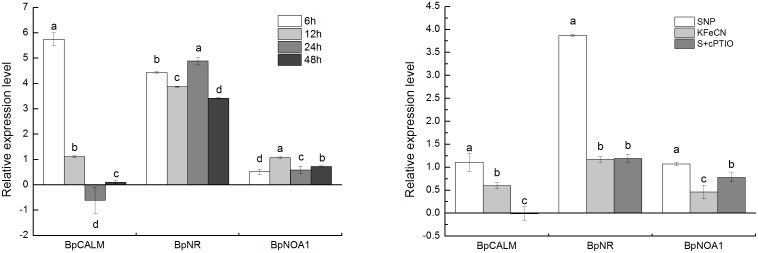
Relative expression levels of genes related to endogenous NO synthesis (*BpNR* and *BpNOA1*) and calcium signaling (*BpCALM*) in groups treated with 1 mM SNP for 6, 12, 24, and 48 h (left). The relative expression levels of genes related to endogenous NO synthesis (*BpNR* and *BpNOA1*) and calcium signaling pathways (*BpCALM*) in the SNP, KFeCN, and SNP + cPTIO (S + cPTIO) groups (right). Different letters represent significant difference (P<0.05).

In the group of genes linked to growth regulation, we found nine genes to be repressed and eight genes to be enhanced. The expression of gibberellin 2-beta-dioxygenase 2 (*G2OX2*), nitrate reductase (*NR*) and NO associated protein1 (*NOA1*), which is similar to nitric oxide synthase (NOS) in animals, was up-regulated in response to SNP exposure. *G2OX2* was involved in gibberellin (GA) synthesis. NO could be produced by the nitric oxide synthase (NOS) pathway and the nitrate reductase (NR) pathway in plants [3], [4]. These results suggest that SNP treatment could influence endogenous NO synthesis. Whereas, the expression of 1-aminocyclopropane-1-carboxylate synthase (*ACC*) was reduced when cells were exposed to NO. This enzyme is typically involved in ethylene synthesis. Of eight repressed genes, we detected three protein kinase genes: nucleoside diphosphate kinase 1 (*NDK1*), mitogen-activated protein kinase kinase kinase 2 (*M3K2*), mitogen-activated protein kinase kinase kinase ANP1 (*ANP1*), which involved in mitogen-activated kinases (MAPK) pathways. The expression of another protein kinase gene, serine/threonine-protein kinase WNK (with no lysine kinase) 4 was up-regulated by SNP treatment. WNK kinase is involved in cell growth and survival signaling, and can inhibit the MEK1/ERK1/2 pathway and cell proliferation [45]. Three genes involved in cell division and proliferation, cell division control protein 45 (*CDC45*), cell division control protein 6 homolog (*CDC6*), and the G2/mitotic-specific cyclin-2 (*CCN2*), were repressed by SNP treatment. Genes involved in cell division were down-regulated to up to 5- to 10-fold when exposed to SNP, indicating that NO affected the cell proliferation process. Meanwhile, rhythmically related genes for transcription factor HY5, rhythmically expressed gene 2 protein (*REG2*), and transcription factor HY5 homolog (*HYH*), which has also been reported to mediate plant responses to hormones, cold, UV-B, and ROS signaling [46], were up-regulated by SNP treatment, indicating that NO was involved in the regulation of plant growth and development. To verify our results from transcriptome analysis, qRT-PCR was performed to quantify the expression levels of *BpCALM* (Calmodulin), *BpNR,* and *BpNOA1* (Fig. 11). The results showed that the expression levels of *BpCALM*, *BpNR*, and *BpNOA1* in the SNP group were significantly higher than those in the KFeCN and SNP + cPTIO group at 12 h. The results of gene expression dynamics showed that three genes were significantly up-regulated after the 6-h SNP treatment. The expression level of *BpCALM* was highest after the 6-h SNP treatment, and significantly lower than the control group at 12 h. The expression of *BpNR* and *BpNOA1* genes were highest after 24 h and 12 h SNP treatment, respectively. The qRT-PCR results indeed indicated that these genes were up-regulated in cells by NO treatment, which suggested exogenous NO was involved in the regulation of endogenous NO signal and Ca^2+^ levels (Fig. 11).

## Conclusions

We examined the gene expression dynamics of *Betula platyphylla* in response to SNP treatment and identified a core set of NO-related transcripts. Taken together, the results from qRT-PCR were consistent with our initial transcriptome analysis. Thus, the combination of transcriptome analysis, gene expression quantification and select enzyme assays indicated that birch cell exposure to NO has a significant effect on many processes including triterpenoid production, carbohydrate metabolism and cell wall biosynthesis.

## Supporting Information

S1 Table Sequences of primer pairs for quantitative real-time RT-PCR assay.
(DOC)
