# Supplementary material for: Genome-Scale Transcriptome Analysis in Response to Nitric Oxide in Birch Cells: Implications of the Triterpene Biosynthetic Pathway
Source: PLoS One. 2014 Dec 31;9(12):e116157. doi: 10.1371/journal.pone.0116157 (PMC4281108; doi:10.1371/journal.pone.0116157)
Supplement: S1 Table — Sequences of primer pairs for quantitative real-time RT-PCR assay. (DOC) [file pone.0116157.s001.doc]

**Table S**1 Sequences of primer pairs for quantitative real-time RT-PCR assay

| **Genes** | **Primer F (forward primer)** | **Primer R (reverse primer)** |
| --- | --- | --- |
| *HMGR* | 5'-CCTCCTTCATCTACCTCCTTGGCT-3' | 5'-CTTGATCATCTCCTCGTCCTCTTC-3' |
| *DXR* | 5'-TGGGGTGATCCAAAAACGAT-3' | 5'-GGAGTCCAAGAAGGAGACGG-3' |
| *SQS* | 5'-GAGTGTGTGCGTGTGTTTCGTAG-3' | 5'-AGAGACCTTGTTGAGCATGGTGT-3' |
| *BPY* | 5'-CTGCTCAGTTCCTTCAAGTC-3' | 5'-TTGCCCATGCAGTATGTACC-3' |
| *CYP716A* | 5'-AAGGAGTCTGTATTTGGGAATGT-3' | 5’- GGCTAGAGACGTTTACATTGGG-3' |
| *CALM* | 5'-TCATCTTTCTCGCCATTAGGTTC-3' | 5'-CTGAGTTCAAGGAGGCATTCAGC-3' |
| *NR* | 5'-TTGTCGGAGTGTATGGCGTCAAACT-3' | 5'-CGCAAACCAATCCCTGAAGAAGAGC-3' |
| *NOA1* | 5'-CTTCTTGTTATGGGTGTGGGG-3' | 5'-CAGTTCTAAGCTGGCGGTGTT-3' |
| *SODF* | 5'-GTCTGCCTTTTTCTCCTTTCCCTCT-3' | 5'-TCCCAATGCTGTTAATCCACTTGTC-3' |
| *SODM* | 5'-AGTCGGTGCTCTTTGCTTTTCC-3' | 5'-TTGCCCACGGCGTCCTGTAG-3' |
| *SODC* | 5'-CAGCATGCACAACAACTGCCCTCC-3' | 5'-TCCACTGGCCCTCATTTCAACCCC-3' |
| *HO* | 5'-TTTCCCAGTCACGCTCCTATTT-3' | 5'-TCATCTCCTCCACGAACCCCTT-3' |
| *SUS2* | 5' TTCAGTTCCTCAACCGTCAC 3' | 5' CTCGTTCTGCTGTATCACCC 3' |
| *CESA8* | 5' AGGCTGGACTATGGAAGATG 3' | 5' GATTTGGACCGAGTTGTGGA 3' |
| *MIOX4* | 5' TGGGCTGTTGTTGGAGACAC 3' | 5' TGCCGTATGCAAAGGATAAA 3' |
| *COMT1* | 5' AACCTCTAGCCCTTCACAGT 3' | 5' GATACGATCCAGCATAACTG 3' |
| *BXL1* | 5' ATTTCAATGCCAAGGTCAGC 3' | 5' AAGAAGGGTCCACAGTCCAA 3' |
| *β-actin* | 5'-TTGCTATCCAGGCTGTTCTC-3' | 5'-TCAAGTTCCTGCTCATAGTCAA-3' |
